# Prevalence of apical periodontitis and frequency of root canal treatments in liver transplant candidates

**DOI:** 10.4317/medoral.19148

**Published:** 2013-05-31

**Authors:** Lizett Castellanos-Cosano, Guillermo Machuca-Portillo, Juan J. Segura-Sampedro, Daniel Torres-Lagares, José López-López, Eugenio Velasco-Ortega, Juan J. Segura-Egea

**Affiliations:** 1 Department of Stomatology, School of Dentistry, University of Sevilla, C/ Avicena s/n, 41009-Sevilla, Spain; 2 Department of General and Digestive Surgery, Virgen del Rocío University Hospital, Sevilla, Spain; 3 Department of Odontostomatology, School of Dentistry, University of Barcelona, Gran Via de les Corts Catalanes 585, 08007-Barcelona, Spain

## Abstract

Aim: The purpose of this study was to investigate the prevalence of apical periodontitis (AP) and endodontic treatment in liver transplant candidates and control healthy subjects. Material and Methods: A descriptive cross-sectional study. Forty two liver transplant candidates (LTC) (study group) and 42 control subjects. Digital panoramic radiographs where used. Periapical status was scored according to the periapical index (PAI). Results were analysed statistically using the Chi-squared test and logistic regression. Results: Radiographic signs of AP in one or more teeth was found in 79% of patients in the study group and in 50% of control subjects (p = 0.008; OR = 3.7; C. I. 95% = 1.4 - 9.5). One or more root-filled teeth (RFT) were found in 19% and 62% of study and control subjects, respectively (p = 0.0001; OR = 0.14; 95% C. I. = 0.05 - 0.38). Among LTC patients 14.7% of the teeth had AP, whereas in the control subjects 4.2% of teeth were affected (p= 0.0002). The percentage of RFT in the study and control groups was 1.5% and 6.8%, respectively (p = 0.0002). Conclusions: Liver transplant candidates have significantly higher prevalence of radiographic periapical lesions and lower frequency of RFT than controls healthy subjects.

** Key words:**Apical periodontitis, endodontics, hepatic cirrhosis, liver disease, liver transplant, oral health, root-canal treatment.

## Introduction

Cirrhosis is the consequence of a sustained wound-healing response to irreversible hepatocellular injury that leads to both fibrosis and nodular regeneration throughout the liver (1). The most common etiologic factors resulting in cirrhosis are hepatitis B, hepatitis C, and excessive alcohol consumption (2). Patients with cirrhosis may frequently present with concurrent etiologic factors, such as chronic hepatitis C with concomitant chronic alcohol consumption. Liver transplantation (LT) is the accepted treatment option for end-stage chronic liver disease (CLD) (1).

In end-stage chronic liver disease (CLD) patients the function of the immune system is compromised (3). Anasarca and malnutrition associated with cirrhosis predispose to poor wound-healing and soft tissue infection (4). Portal venous shunts contribute to systemic spread of infection by bypassing the hepatic filtration (5). Therefore, infections are a frequent cause of morbidity and mortality among patients with CLD and, after LT, immunosuppression predisposes much more to infections (6). It has been estimated that 60 to 80% of liver transplant recipients develop an infection that compromises their survival (7), being infections a major cause of mortality among immunosuppressed post-LT patients (8).

Human oral cavity may act as a major, not well-known, source of pathogens to induce clinically important postoperative mixed infections, including developing of high-risk graft complications (9). A survey of U.S. organ transplant centers, conducted between 2003 and 2004, found that among 294 respondents, 28 (9%) reported that they had encountered 1 or more incidents of sepsis from a dental source in a transplant recipient (10). In addition, 34 centers (11%) experienced 1 or more episodes of a dental infection prior to transplantation that required cancellation or postponement of the surgery (9). Helenius-Hietala J et al. (11) compared the occurrence of post-transplant infections between the acute or subacute liver failure patients who either had or had not undergone dental examination and treatment for dental infectious foci before LT and found a significantly higher incidence of post-transplant infections in the group without dental treatment than in the group with dental treatment. Therefore, an oral examination has been proposed as a prerequisite prior to LT aiming to detect and to eliminate the possible oral infection focus in order to reduce bacteremia and eventually morbidity (12,13). Moreover, liver transplantations are performed for complications resulting from viral hepatitis or alcoholic cirrhosis, both states frequently associated with lifestyles and behaviors that contribute to dental neglect and untreated dental disease (7). Indeed, patients with CLD, particularly those with hepatitis C virus infection or alcoholic liver disease, have been shown to display poor state of oral health (14,15). However, there is limited data on how dental disease impacts post-transplant outcomes.

Amongst oral infectious diseases, apical periodontitis (AP) is, after caries, the most prevalent oral infection both in USA (16), and Europe (17,18). AP is a focal infection characterized by a radiolucent lesion around the apex of a tooth caused by bacterial infection of the pulp canal system. Endodontic therapy (i.e., root-canal treatment) is the elective treatment for teeth with AP which must be preserved. However, radiolucent periapical lesions (RPL) can be observed in 63% of root-filled teeth (17). Diabetes mel-litus (19,20) and smoking have been identified as risk factors for AP (21,22).

Although it has been reported that oral health attributes in candidates for liver transplantation (i.e., gingivitis, dental plaque, dental caries, periodontal disease, edentulism, and xerostomia) were similar to those seen in the general population (7), no investigation has studied the frequency of AP, identified as RPL, and root-canal treatment amongst patients with CLD candidates to LT.

The purpose of the present study was to investigate the prevalence of RPL and the frequency of endodontic treatment in liver transplant candidates and control healthy subjects. The null hypothesis was that liver transplant candidates have the same frequency of RPL and endodontic treatment that control subjects.

## Material and Methods

The experiments were undertaken with the understanding and written consent of each subject and according to the World Medical Association Declaration of Helsinki. The protocol was approved by the Ethical Committee of the “Virgen del Rocío” University Hospital, Sevilla, Spain. Both liver transplant candidates (LTC) and control subjects were asked to voluntarily participate in the study. Each subject signed a consent form after being advised of the nature of the study.

Liver transplant candidates (LTC) included in the study group were recruited among patients who were listed for liver transplantation at the “Virgen del Rocío” University Hospital (Sevilla, Spain) between the years 2008 and 2011. Inclusion criteria were as follows: patients older than 18 years, having at least 8 remaining teeth, who agreed to a radiographic examination. Exclusion criteria encompassed patients younger than 18 years old, having less than 8 remaining teeth, or who did not agree a radiographic examination. A total of 42 liver transplant candidates (LTC), 30 men and 12 women (59.1 ± 8.6 years), that agreed and met the inclusion/exclusion criteria constituted the “study group”. An additional 42 subjects, 30 men and 12 women (59.3 ± 8.7 years), reporting no history of liver disease nor alcoholism and who also met the inclusion/exclusion criteria of the study, constituted the “control group”. Control participants were recruited from patients of the same health district seeking for the first time routine dental care (not emergency care) at the Dental Clinic of the University of Sevilla, Spain, between the years 2008 and 2011.

-Radiographic examination

Radiographic periapical status was diagnosed on the basis of examination of digital panoramic radiographs of the jaws. Two trained radiographic technicians, with over ten years of experience, took the panoramic radiographs using a digital ortho-pantomograph machine (Promax®, Planmeca, class 1, type B, 80 KHz, Planmeca, Helsinki, Finland).

-Radiographic evaluation

The periapical status was assessed using the “Periapical Index” (PAI) , as described previously . Briefly, each of the roots was categorized as: 1- Normal periapical structure; 2- Small changes in bone structure; 3- Changes in bone structure with some mineral loss; 4- Periodontitis with well-defined radiolucent area; and 5- Severe periodontitis with exacerbating features. Each category used in the PAI represents a step on an ordinal scale of registration of periapical infection.

A score greater than 2 (PAI ? 3) was considered to be a sign of periapical pathology. The worst score of all roots was taken to represent the PAI score for multi-rooted teeth. Teeth were categorized as root-filled teeth if they had been filled with a radiopaque material in the root canal(s).

The following information was recorded on a structured form for each subject: (a) number of teeth present; (b) number and location of teeth having identifiable periapical lesions, (c) number and location of root-filled teeth, and (d) number and location of root-filled teeth having identifiable periapical lesions.

-Observers’ calibration

Three observers with extensive clinical experience in endodontics examined the radiographs. Before evaluation, the observers participated in a calibration course for PAI system, which consisted of 100 radiographic images of teeth, some root-filled and some not, kindly provided by Dr. Ørstavik. Each tooth was assigned to 1 of the PAI scores by using visual references (also provided by Dr. Ørstavik) for the 5 categories within the scale. After scoring the teeth, the results were compared to a “gold standard atlas”, and a Cohen Kappa was calculated (0.79 – 0.85).

After the PAI calibration, intra-observer reproducibility was evaluated for each examiner. Every observer scored the panoramic radiographs of 20 patients (10 of each group, randomly selected). Then, one month after this first examination, the observer was recalibrated in the PAI system and repeated the scoring of the radiographs of the same 20 patients. The intra-observer agreement test on PAI scores on the 20 patients produced a Cohen’s Kappa ranging 0.84 - 0.91.

Finally, intra-observers reproducibility was also determined comparing the PAI scores on the 20 radiographs provided by each observer. The agreement test produced a Cohen’s Kappa ranging 0.83 - 0.92. The Cohen’s Kappa for inter-observers variability ranged 0.76 - 0.83. The consensus radiographic standard was the simultaneous interpretation by three examiners of the panoramic radiograph of each patient (25,26).

-Statistical analysis

The minimal sample size was calculated for the comparison of proportions in two independent samples, taking into account a two-sided significance level of 5% (? = 0.05, Z? = 1.960), a 80% power (? = 0.20, Z? = 0.842) to detect a significant difference, and a hypothesized difference between the proportions of the two groups of 30 points (prevalence of AP reported previously in Spain~ 50% (17,27), hypothesized prevalence of AP in the study group = 80% calculated from the preliminary results of a pilot study). The calculated minimal sample size (n = 38) was increased to 42 in order to more accurately reflect the prevalence of radiolucent periapical lesions.

Raw data were entered into Excel (Microsoft Corporation, Redmond, WA). All analyses were done in an SPSS environment (Version 11; SPSS, Inc, Chicago, IL). The Student t test, ?2 test, and logistic regression analysis were used to determine the significance of differences between groups. Data are reported as mean ± standard deviation.

## Results

There were no significant differences between study and control groups in age, gender and smoking habits ( Table 1). In the study group, 57.1% of patients were diabetics, whereas the proportion of diabetic subjects in the control group was 21.4% (p < 0.01). Alcohol consumption was present in 21.4% and 40.5% of LTC and controls, respectively (p < 0.01). The median MELD (Model for End-Stage Liver Disease) score in LTC was 14.5 (range = 6 – 25).

**Table 1:**
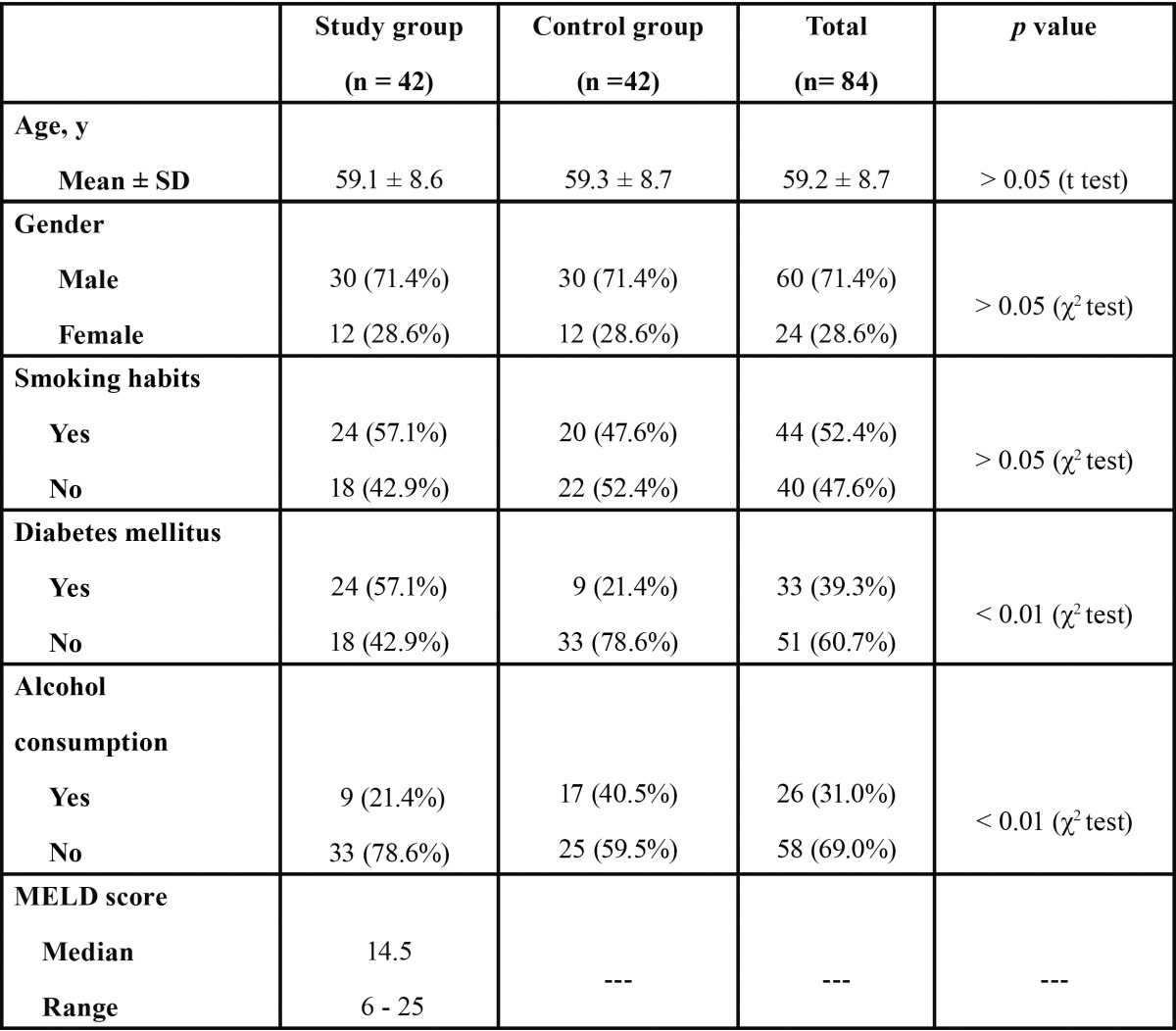
Characteristics of end-stage chronic liver disease patients (study group) and control healthy subjects.

The distribution of the analyzed variables in the two groups is shown in  Table 2. The average number of teeth per subject was 18.6 ± 5.9 and 21.6 ± 6.1 in the study and control subjects, respectively (p < 0.05). RPL in 1 or more teeth was found in 33 LTC patients (78.6%) and in 21 control subjects (50%) (p < 001; OR = 3.7; 95% C.I. 1.4 - 9.5). The average number of teeth with RPL per patient was 2.7 ± 2.7 and 1.0 ± 1.3 in LTC and control subjects, respectively (p < 0.01). Root-filled teeth were found less fre-quently in the study group. The average number of root-filled teeth per subject was 0.3 ± 0.7 in LTC and 1.6 ± 2.0 in controls (p < 0.01). One or more root-filled teeth were found in 19.0% (n = 8) and 61.9% (n = 26) of study and control subjects, respectively (p < 0.01). The average number of root-filled teeth with RPL per subject was 0.29 ± 0.6 in LTC and 0.5 ± 1.0 in control subjects (p < 0.05).

**Table 2:**
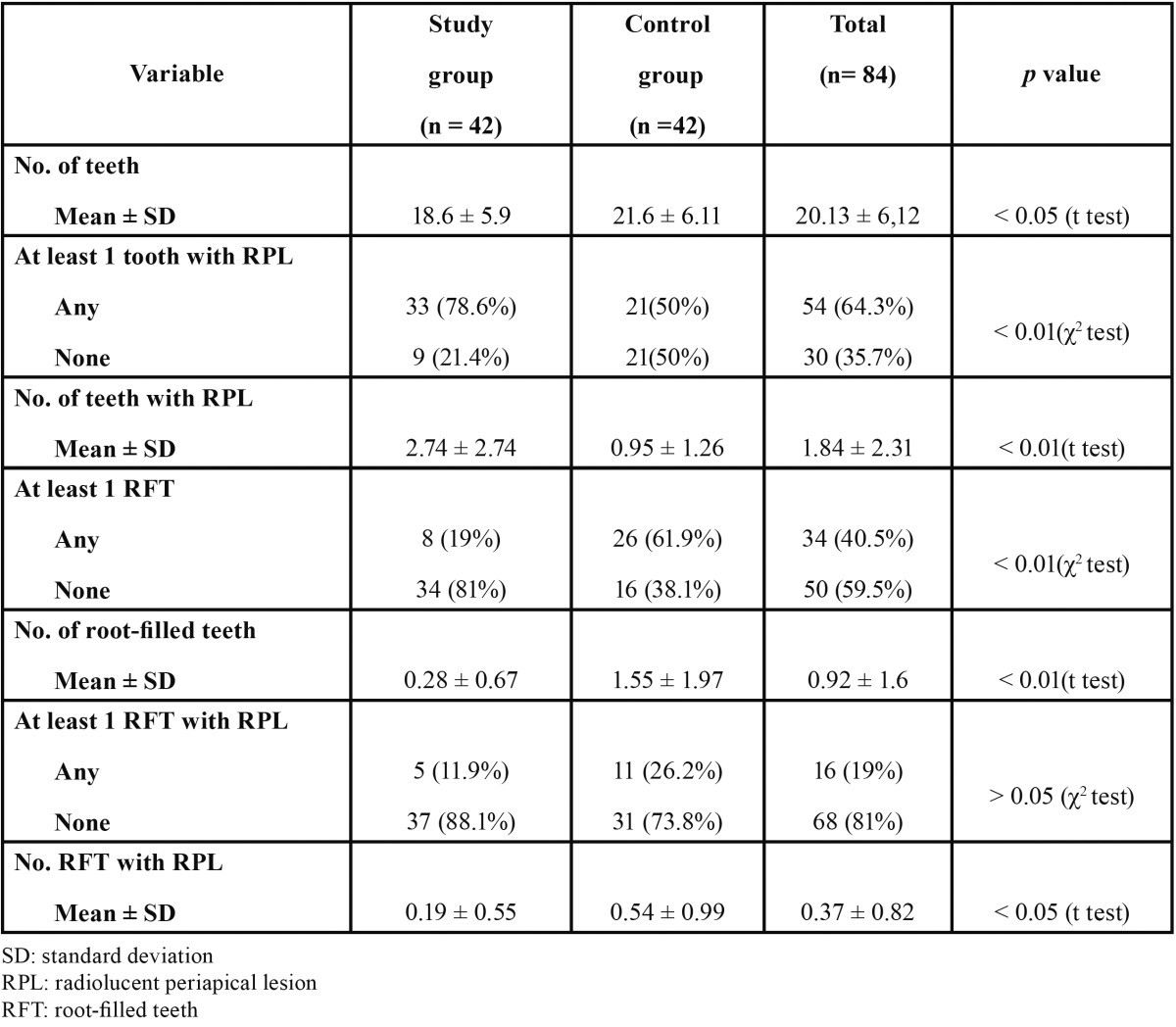
Distribution of the analyzed variables amongst end-stage chronic liver disease patients (study group) and control healthy subjects.

Multivariate logistic regressions were run with age, gender (male/female), smoking habits (yes/no), diabetes (present/absent), alcohol consumption (present/absent), number of teeth, endodontic treatment (at least 1 root-filled tooth), and liver transplant candidate (yes/no) as explanatory/independent variables, and at least 1 tooth with RPL (yes/no) as dependent variable and out-come ( Table 3). In the multivariate analysis including all the above factors as covariates, liver transplant candidate status remained associated significantly (p = 0.0029; OR = 7.6; C.I. 95% 2.0 - 28.8) to the presence of radiographically diagnosed apical periodontitis, indicating that LTC have RPL with higher likelihood than control subjects. Age (p = 0.03) and endodontic status (p = 0.03) were also significantly associated to periapical status. Smoking habits, diabetes and alcohol consumption were not associated to the presence of RPL (p > 0.05).

**Table 3:**
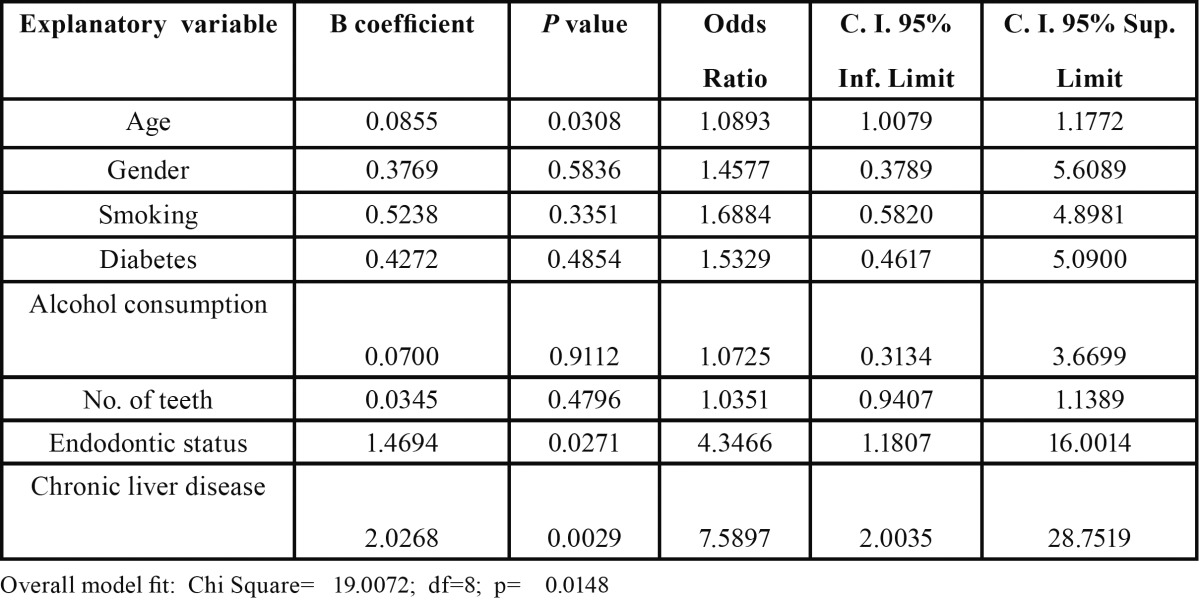
Multivariate logistic regression analysis of the influence of the explanatory variables age, gender, smoking (0 = no, 1 = yes), diabetes (0 = no, 1 = yes), alcohol consumption (0 = no, 1 = yes), teeth number, endodontic status (0 = none, 1 = one or more root-filled teeth), and chronic liver disease (0 = no, 1 = yes), on the dependent variable “periapical infection” (0= none, 1= one or more teeth with radiolucent periapical lesion).

## Discussion

This cross-sectional study aimed to investigate the prevalence of RPL using the PAI index, in patients with CLD candidates to liver transplant and control healthy subjects. Results reveal that LTC have significantly higher prevalence of RPL (78.6%) than control subjects (50%) (p < 0.01; OR = 7.6; C.I. 95% 2.0 - 28.8). Moreover, the average number of teeth with RPL per patient was nearly three times that in control subjects (p = 0.0002). Epidemiologic studies have reported that 34% – 61% of Spanish adults have AP17,19. Thus, the prevalence of RPL in LTC can be considered high.

Several studies have shown an association between dental infections and general health. However, a direct causative relationship has not been established (28). Although the role of chronic apical periodontitis and endodontic therapy in the development of adverse systemic outcomes has not been thoroughly explored, several investigations suggest their association with type II diabetes (19,29) and coronary heart disease (30,31). Among the multiple causes of post-transplantation infection that are cited in the literature, however, dental sources have rarely been implicated (32,33).

Although periapical infectious process produces a variety of local tissue responses with the likely purpose to confine and limit the spreading of the infectious elements, apical periodontitis may not exclusively be a local phenomenon (34). In its non-balanced acute stage, spreading of the infection and the inflammatory process to nearby tissue compartments is possible and may bring about severe, but fortunately rare, fatal inflammatory conditions. Moreover, considering the increasing awareness of a potential relationship between persistent, inflammatory disorders of the oral cavity and disease conditions in other organs of the body, acute and chronic manifestations of AP may also be implicated (35).

Patients with CLD, particularly those with hepatitis C virus infection or alcoholic liver disease, have been shown to display poor state of oral health . In previous studies alcohol and hepatitis C cirrhotic patients had the lowest number of teeth when compared with healthy controls (9,10). The results of the present study, i.e. a low number of teeth in the study group (p = 0.0311; OR, 0.92), are in agreement with these previous findings. The poor oral health status in LTC can be attributable not only to poor oral hygiene but also to inadequate dental care (10,12). Other physical, behavioral, and/or social comorbidities among LTC that could contribute to untreated dental disease, as well as tooth loss, include their older age, education level, preoccupation with medical issues, use of medications that reduce salivary flow, lack of motivation, anxiety and/or depression, poor health behaviors and cognitive loss (37,38). These factors could also have added impact if the patients have a prolonged period on the waiting list (7). Lins et al. (2001) have recently concluded that poor oral health status observed in most CLD patients may represent a source of systemic infections before and after liver transplantation. Treatment of such lesions was feasible in the majority of the patients and seemed to be associated with a reduction in mortality (39).

The percentage of subjects having at least 1 root-filled tooth varied significantly in LTC (19.0%) compared to control subjects (61.9%) (p = 0.0001; OR, 0.1448). This low frequency of endodontic therapy could indicate and inadequate dental care in LTC (7). Root canal treatment, i.e. endodontic therapy, is the elective treatment of apical periodontitis. The elimination of infected pulp and the protection of the decontaminated tooth from future microbial invasion avoid the leakage of antigens to periapical tissues, allowing apical and periapical wound healing. New protocols in dental care in LTC patients before transplantation surgery must be established in order to treat all the teeth with apical periodontitis, either by endodontic therapy, and either by tooth extraction. Root canal treatment has the advantage that maintains the tooth and decreases the need of performing dental surgical procedures in LTC, avoiding the risk of hemorrhagic complications and delayed wound healing (40). However, teeth with obvious infections, and all non-restorable teeth, must be extracted prior to transplantation even though the effect of the practice in preventing septic episodes remains controversial (41).

When root canal treatment cannot be performed and teeth must be extracted, future prosthetic solution must be given. Liver transplant candidates with various degrees of edentulism may request dental implant treatment. Uncontrolled late healing of the wound and oral infection could ruin the transplanted organ and even be fatal (42). Consequently, immunocompromise after organ transplantation has been generally regarded to be a contraindication for dental implants in these patients. However, Gu et al. (43) found in liver transplants recipients no relevant side effect, such as infection or peri-implantitis, which could demonstrated the predictability and safety of dental implant therapy in liver transplant patients.

## Conclusion

Liver transplant candidates have significantly higher prevalence of radiographic periapical lesions and lower frequency of root-filled teeth than controls healthy subjects. Taking into account that dental infections may increase susceptibility to infections before and after liver transplantation, apical periodontitis must be actively sought and treated accordingly in liver transplant candidates.
